# Using Experimental Evolution to Correct Mother-Daughter Separation Defects in Brewing Yeast

**DOI:** 10.17912/micropub.biology.001962

**Published:** 2026-02-13

**Authors:** Lauren M Ackermann, Amanda Ro, Barbara Dunn, Ryan Moore, Greg Doss, Joseph O Armstrong, Maitreya J Dunham

**Affiliations:** 1 Genome Sciences, University of Washington, Seattle, Washington, United States; 2 Imperial Yeast, Portland, Oregon, United States

## Abstract

Brewers have domesticated many strains of *Saccharomyces cerevisiae* with traits complementary to different beers, but these strains may also harbor undesirable characteristics. One example is the mother-daughter separation defect (MDSD) which is present in London Ale III, a popular brewing strain, and causes cells to form large clusters. MDSDs can be caused by mutations to several genes, making targeted genetic approaches to reduce MDSDs challenging. We passaged three populations for over 200 generations to generate strains with reduced MDSD, demonstrating how experimental evolution can be used to select against undesirable traits in industrial yeast strains.

**Figure 1. Improving MDSDs with experimental evolution:**
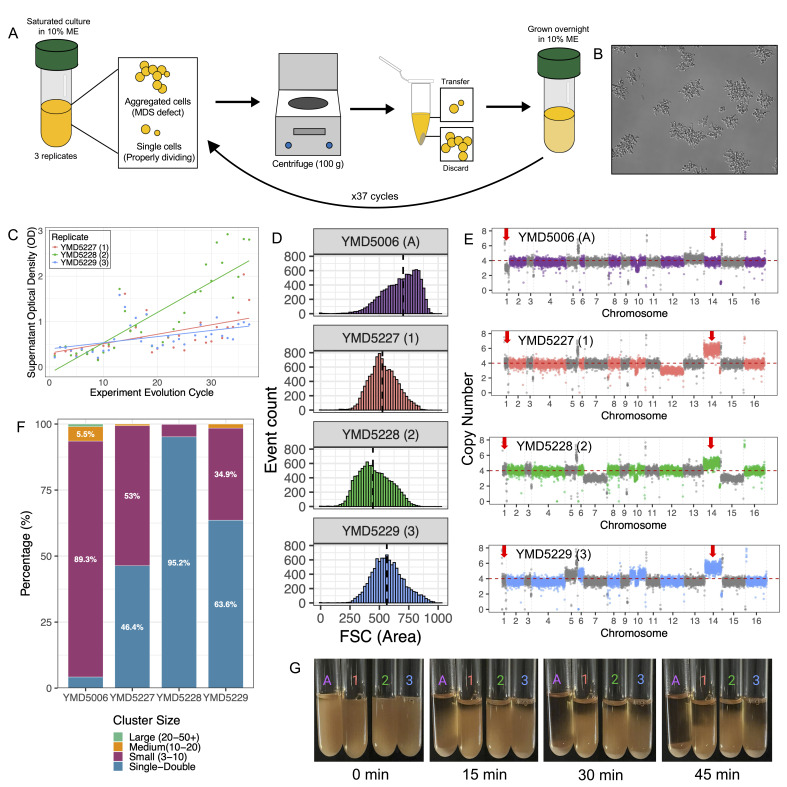
**A.** A schematic of our experimental evolution cycle, performed daily with three replicate populations and grown overnight at 30°C in 10% Malt Extract. **B.** Microscope image at 300x magnification of an overnight culture from the ancestral strain. **C.** Plot showing supernatant optical density (OD600) over time, measured by experimental evolution cycles. Each replicate is indicated by a different color: red, green, and blue representing the 1st, 2nd, and 3rd replicate, respectively. A line of best fit in the same color is plotted to demonstrate the general trend in optical density. **D.** Histograms demonstrating forward scatter (FSC, a proxy for cell and cluster size) of an individual clone isolated from the ancestor and each replicate, measured with flow cytometry. Event count is shown on the y-axis, while FSC-A is shown on the x-axis. The median cell/cluster size is approximated and indicated by the dashed black line on each histogram. Purple, red, green, and blue correspond to clones from the ancestor (YMD5006), replicate 1 (YMD5227), replicate 2 (YMD5228), and replicate 3 (YMD5229), respectively. **E.** Copy number plots of YMD5006, YMD5227, YMD5228, and YMD5229 generated with Illumina sequencing using the read depth across 1000 base pair rolling windows. **F.** Bar graph showing the percentage of cells or cell clusters that fall into different size categories in samples taken from the ancestor, YMD5227, YMD5228, and YMD5229. The smallest category (single-double) represents either one single cell or two cells clustered together, and the largest category (large) represents 20 or more cells in a cluster. **G.** Qualitative settling assay demonstrating settling rates over time of three replicate clones. Culture tubes are shown in 15-minute intervals and different colors represent the ancestor clone and three replicate clones.

## Description

*Saccharomyces cerevisiae* replicate through a process known as budding, where a mother cell undergoes cytokinesis to form a daughter cell, which emerges as a bud. During this process, the cytoplasm is divided by the contraction of the actomyosin ring and by the formation of a specialized wall between the mother and bud called the septum. Cell division is successful when the septum is completely broken down, allowing the mother and daughter cells to fully separate (Weiss 2012). When the septum is unable to fully break down, mother and daughter cells remain connected leading to a mother-daughter separation defect (MDSD). MDSDs cause dividing cells to form large chains or clusters that are joined together at bud necks. These clustered cells are sometimes referred to as “snowflake yeast” due to their appearance (Ratcliff et al. 2012). Brewing strains with MDSDs accumulate large amounts of floating foam during active fermentation, requiring specialized release valves and/or more starting headspace (less wort) in the fermenter, resulting in lower fermentative yields. It is possible that the “snowflake” cell clusters could be responsible for this behavior.

Despite this disadvantage, many brewers still choose to use strains with MDSDs due to other desirable traits these strains hold. The goal of this project was to correct MDSDs in a widely used and commercially available brewing strain, London Ale III, the most common strain used to make New England style (Hazy) IPAs. To verify that the clusters in our starting strain were primarily due to MDSD and not flocculation, we washed cells with EDTA, a chelating agent that inhibits flocculation, as previously described (Hope et al. 2017). We did not observe a major change in clustering behavior, indicating that cluster formation in the ancestor is not driven by flocculation. We also used Calcofluor White to stain bud scars in the ancestor strain and observed that clustered cells were connected at the bud neck, further verifying that cluster formation is driven by MDSD. Correcting MDSDs in a targeted way is challenging because MDSDs can be caused by mutations to multiple genes, for example, *AMN1* (Fang et al. 2018) and *ACE2* (Voth et al. 2005, Ratcliff et al *.* 2015). Moreover, London Ale III is tetraploid, which further complicates a targeted genetic approach. To overcome this challenge, we sought to ameliorate MDSD in the London Ale III strain by using experimental evolution to select against aggregating cells, as has been previously described (Amorosi 2020, Ratcliff et al. 2012, Galeota-Sprung et al. 2026).

In laboratory culture, large, clustered aggregates are less buoyant and settle faster than single cells. In our experimental evolution protocol, we took advantage of this property by using gentle centrifugation (100 x g) for a short duration (20 seconds) to enrich smaller clusters and single cells in the supernatant. We then transferred the supernatant into fresh media to complete the cycle ( Fig. 1A ). To emulate brewing conditions, evolutions were performed using 10% Malt Extract (ME). We repeated this cycle 37 times (approximately 200 generations) for three biological replicates, then isolated clones from each of the three evolved populations to generate novel strains (YMD5227, YMD5228, and YMD5229). We observed by microscopy that large cell clusters decreased in frequency in all three of our evolved clones, while the frequency of single cells and smaller clusters increased compared to the ancestor (representative image of ancestor culture shown in Fig. 1B ). We confirmed these observations both qualitatively and quantitatively with three orthogonal measurements.

First, we measured the optical density of the supernatant ( Fig. 1C ) during each experimental cycle and found that it increased over time in all three of our replicates, suggesting a higher proportion of single and small clusters. After 23 evolution cycles, we began taking optical density measurements of the overnight culture before centrifugation. We found that over time, the ratio of supernatant OD to overnight culture OD increased, validating that single and small clusters increased in our replicates. We also conducted a qualitative settling assay, where tubes containing overnight cultures of our three evolved clones remained turbid for longer than an overnight culture of the ancestral strain. After 45 minutes, we found the ancestral strain completely settled, while the evolved clones remained cloudy ( Fig. 1G ). We performed this assay on four occasions and found that our evolved replicates settled notably slower than the ancestor.

Next, we quantitatively assessed the size distribution of the evolved clones (YMD5227, YMD5228, and YMD5229) using flow cytometry to measure the intensity of forward light scattering, which correlates with cell or cluster size. We generated histograms of forward scatter intensity for the three evolved strains and compared them with the ancestor strain. All three evolved strains had a lower median forward scatter than the ancestor ( Fig. 1D ), indicating that the evolved strains form smaller clusters and contain more single cells than the ancestor. Finally, we used a hemocytometer to examine overnight cultures of YMD5006, YMD5227, YMD5228, and YMD5229 grown in 10% ME. We observed various-sized clusters of cells with characteristic snowflake shapes in the ancestor and the evolved clones; however, the ancestor had very few single or doublet cells, while all evolved strains had many more of these. In fact, the proportion of single-double clusters out of the total number of clusters increased from 4% in the ancestor to 95% in YMD5228, 64% in YMD5229, and 46% in YMD5227 ( Fig. 1F ). These results indicate that we successfully selected against cells with MDSDs and generated strains that feature more single cells and fewer large clusters. We identified from our settling assay, cytometric analysis, and cluster size counts that the evolved strain YMD5228 settled the slowest and had the smallest average cluster size.

To uncover the genetic basis of the improvement in MDSD in our evolved clones, we performed whole genome sequencing on the evolved strains and the ancestral strain. We analyzed the sequences of the evolved strains and searched for single nucleotide polymorphisms (SNPs) or copy number variation that may explain the improved MDSD in our evolved strains. We first looked specifically for mutations in *ACE2* , *AMN1* , *CTS1* , *DSE4* , *DSE2* , *SUN4* , *DSE1* , *IZH4,* and *SCW11* , genes known to be associated with the snowflake phenotype (Ratcliff et al *.* 2015). Across all evolved strains, we did not find new SNPs in these genes, only changes to existing allele frequencies (see below). We then looked at genes involved in *AMN1* negative selection: *STE11* , *RIM8* , *RIM13* , *RIM20* , and *RIM101* (Galeota-Sprung et al. 2026). Again, we did not find meaningful and novel SNPs in our evolved clones, only to preexisting allele frequencies. We also did not find evidence for loss of heterozygosity, a hallmark of mitotic recombination (Zhu et al. 2025).

To explore whether changes to whole chromosome copy numbers could account for the improvement in MDSD, we generated copy number plots of the ancestor and evolved strains using the read depth across 1000 base pair rolling windows ( Fig. 1E ). We found that each evolved strain experienced a copy number increase of chromosome 14, as well as of chromosome 1. Chromosome 1 increases from three copies in the ancestral strain to four copies in all of the evolved strains. Chromosome 14 increases from four copies in the ancestral strain to five copies in YMD5228 and six copies in YMD5227 and YMD5229. Allele frequencies of SNPs in chromosome 1 were consistent with three copies for the ancestor, but four copies for the evolved clones. We saw similar patterns in allele frequencies of SNPs located on chromosome 14, consistent with six copies in YMD5227 and YMD5229, and five copies in YMD5228. Different copies of each chromosome were amplified in each clone. We observed other abnormal chromosome copy numbers–or aneuploidies–in the evolved strains; however, only the increases in copy number of chromosomes 1 and 14 are shared between all three evolved strains. Two of our candidate genes, *DSE4* and *SUN4* , are located on chromosome 14. Thus, the increase in copy number to either (or both) chromosomes 1 and 14 may play a role in improving MDSD in this strain.

Using experimental evolution, we successfully corrected the MDSD in London Ale III. The strain YMD5228, which showed the most improvement to MDSD, is now commercially available to brewers. In the future, we want to identify specific causal genes that were involved in driving a non-aggregation phenotype in these evolved strains. We hope to identify which genes on chromosomes 1 and 14 might affect the phenotype, enabling a more targeted approach to engineering other strains. This could provide greater insight into abnormal yeast cell separation and the genotypic changes driving this phenotype in a tetraploid brewing strain.

## Methods

*Strains/Media:*

10% ME media was made by adding 50g of dry malt extract (Briess CBW Pilsen Light Dry Malt Extract #5760) to a sterile jug, then topping off with distilled water to a final volume of 500mL. This mixture was autoclaved, then decanted to remove large particulates. Finally, media was vacuum filtered to remove any remaining particles and ensure sterility.

*Experimental Evolution Cycle:*

We performed a modified method to one previously described (Amorosi 2020, Ratcliff et al. 2012). Overnight cultures were vortexed and 1.5mL was transferred to a sterile Eppendorf tube. Tubes were briefly vortexed, then centrifuged at 100 x g for twenty seconds. 1mL of supernatant was immediately pipetted into culture tubes containing 4mL of 10% ME (see Fig. 1A ). Pipetting 1mL of supernatant was done carefully by moving down from the top of the tube in order to capture the smallest clusters and avoid the pellet. Culture tubes were then vortexed and placed on a roller drum at 30°C to grow overnight. Cells were propagated daily in this manner for 37 evolution cycles (approximately 200 generations).

*Supernatant Optical Density Measurements:*

Optical density (OD600) measurements were taken using BioRad SmartSpec™ 3000. Overnight cultures of each replicate were vortexed, then were transferred to a 1.5mL Eppendorf tube. Tubes were vortexed, then centrifuged at 100 x g for twenty seconds. 1mL of the supernatant was pipetted by carefully moving down from the top of the tube then transferred into a clean cuvette. The spectrophotometer was blanked using 1mL of 10% ME. After 23 evolution cycles, optical density measurements were also taken of the overnight culture in a similar manner. Mixtures were diluted as needed to remain in the linear range of the spectrophotometer (<1.0 OD).

*Generation Number Estimation:*

Generations were defined as a doubling of the estimated cell mass, as measured by optical density. The generations between each cycle were estimated using OD600 of the transferred supernatant and the overnight culture using the following equation:

Generations = log _2_ ​(OD _overnight_ /​OD _supernatant_ ​​)

*Settling Assay:*

Culture tubes containing the evolved clones and the ancestor were thoroughly vortexed, clamped onto a lab stand, and imaged every 15 minutes for a total of 90 minutes. Images were taken with an iPhone 13 clamped at the same position over the course of the assay.

*Flow Cytometry:*

Overnight cultures of each strain were diluted 1:100 and forward scatter intensity was measured on a Sony SA3800 flow cytometer. At least 10,000 events were recorded per sample.

*Illumina sequencing:*

Genomic DNA was isolated from the ancestor and a single clone from each of the three evolved populations using Hoffman/Winston phenol chloroform extraction (Hoffman and Winston 1987). Genomic DNA was purified using the Zymo Research DNA Clean & Concentrator-5. The purified DNA was then prepared for Illumina short read sequencing using the Nextera XT DNA Library Preparation Kit. Each sample received >10 million paired end reads. Determination of indels, copy number variation, and SNPs followed a modified protocol as previously described (Taylor et al. 2022). Short read sequences were aligned to the *Saccharomyces cerevisiae* reference genome (SacCer3) then copy number plots were generated by analyzing read depth across 1,000 base pair sliding windows, normalizing to read depth across all chromosomes, and normalizing to the ploidy of the strain (Large et al. 2020). Sequencing data is available in the NCBI Sequence Read Archive (SRA) under BioProject PRJNA1358295.

## Reagents

**Table:** 

**Strain**	**Species**	**Source/Reference**
Juice (London Ale III)	*Saccharomyces cerevisiae*	Imperial Yeast
